# 4-[2-(Pyridin-1-ium-4-yl)eth­yl]pyridin-1-ium bis­(2,6-di­nitro­benzoate)

**DOI:** 10.1107/S1600536813026810

**Published:** 2013-10-02

**Authors:** Hadi D. Arman, Tyler Miller, Edward R. T. Tiekink

**Affiliations:** a Department of Chemistry, The University of Texas at San Antonio, One UTSA Circle, San Antonio, Texas 78249-0698, USA; b Department of Chemistry, University of Malaya, 50603 Kuala Lumpur, Malaysia

## Abstract

The asymmetric unit of the title salt, C_12_H_14_N_2_^2+^·2C_7_H_3_N_2_O_6_^−^, comprises half a 4-[2-(pyridin-1-ium-4-yl)eth­yl]pyridin-1-ium dication, being disposed about a centre of inversion, and a 2,6-di­nitro­benzoate anion, in a general position. In the anion, the carboxyl­ate group is inclined to the benzene ring [dihedral angle = 85.45 (9)°], whereas near-coplanar and twisted arrangements are found for the nitro groups [O—N—C—C torsion angles = 179.80 (14) and 20.2 (2)°]. In the crystal, three-component aggregates sustained by charge-assisted N^+^—H⋯O^−^ hydrogen bonds are found and these are consolidated into a three-dimensional architecture by C—H⋯O and π–π [inter-centroid distances = 3.6796 (14) and 3.7064 (14) Å] inter­actions.

## Related literature

For the 2:1 salts of 2,6-di­nitro­benzoate with isomeric *n*-({[(pyri­din-1-ium-*n*-ylmeth­yl)carbamo­yl]formamido}­meth­yl)pyridin-1-ium, *n* = 2, 3 and 4, and for the structure of 2,6-di­nitro­benzoic acid, see: Arman *et al.* (2013 ▶).
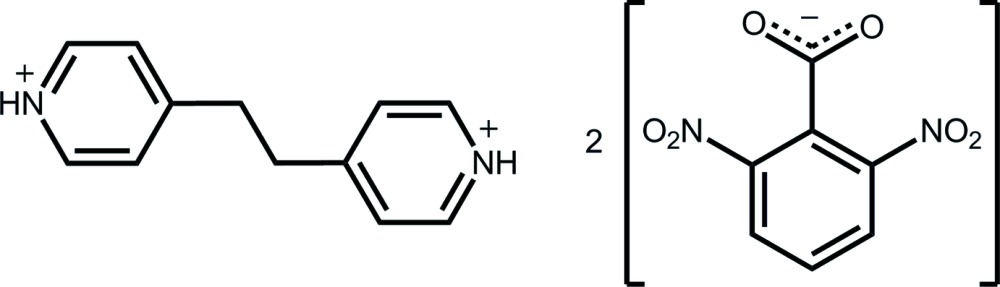


## Experimental

### 

#### Crystal data

0.5C_12_H_14_N_2_^2+^·C_7_H_3_N_2_O_6_^−^
*M* *_r_* = 304.24
Triclinic, 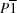

*a* = 6.6916 (12) Å
*b* = 8.3690 (17) Å
*c* = 12.358 (3) Å
α = 88.809 (12)°
β = 76.322 (8)°
γ = 72.193 (9)°
*V* = 639.2 (2) Å^3^
*Z* = 2
Mo *K*α radiation
μ = 0.13 mm^−1^
*T* = 98 K
0.36 × 0.12 × 0.07 mm

#### Data collection

Rigaku AFC12/SATURN724 diffractometer
4421 measured reflections
2915 independent reflections
2538 reflections with *I* > 2σ(*I*)
*R*_int_ = 0.035

#### Refinement

*R*[*F* ^2^ > 2σ(*F* ^2^)] = 0.050
*wR*(*F* ^2^) = 0.124
*S* = 1.07
2915 reflections
202 parameters
1 restraint
H atoms treated by a mixture of independent and constrained refinement
Δρ_max_ = 0.33 e Å^−3^
Δρ_min_ = −0.28 e Å^−3^

### 

Data collection: *CrystalClear* (Molecular Structure Corporation & Rigaku, 2005 ▶); cell refinement: *CrystalClear*; data reduction: *CrystalClear*; program(s) used to solve structure: *SHELXS97* (Sheldrick, 2008 ▶); program(s) used to refine structure: *SHELXL97* (Sheldrick, 2008 ▶); molecular graphics: *ORTEPII* (Johnson, 1976 ▶) and *DIAMOND* (Brandenburg, 2006 ▶); software used to prepare material for publication: *publCIF* (Westrip, 2010 ▶).

## Supplementary Material

Crystal structure: contains datablock(s) general, I. DOI: 10.1107/S1600536813026810/hg5351sup1.cif

Structure factors: contains datablock(s) I. DOI: 10.1107/S1600536813026810/hg5351Isup2.hkl

Additional supplementary materials:  crystallographic information; 3D view; checkCIF report

## Figures and Tables

**Table 1:** Hydrogen-bond geometry (Å, °)

*D*—H⋯*A*	*D*—H	H⋯*A*	*D*⋯*A*	*D*—H⋯*A*
N3—H3n⋯O1	0.90 (1)	1.64 (2)	2.5240 (19)	166 (2)
C8—H8⋯O6^i^	0.95	2.50	3.436 (2)	169
C11—H11⋯O2^ii^	0.95	2.52	3.377 (2)	150
C12—H12⋯O4^iii^	0.95	2.46	3.118 (2)	126

Symmetry codes: (i) 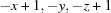; (ii) 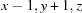; (iii) 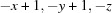.

**Fig. 1.**
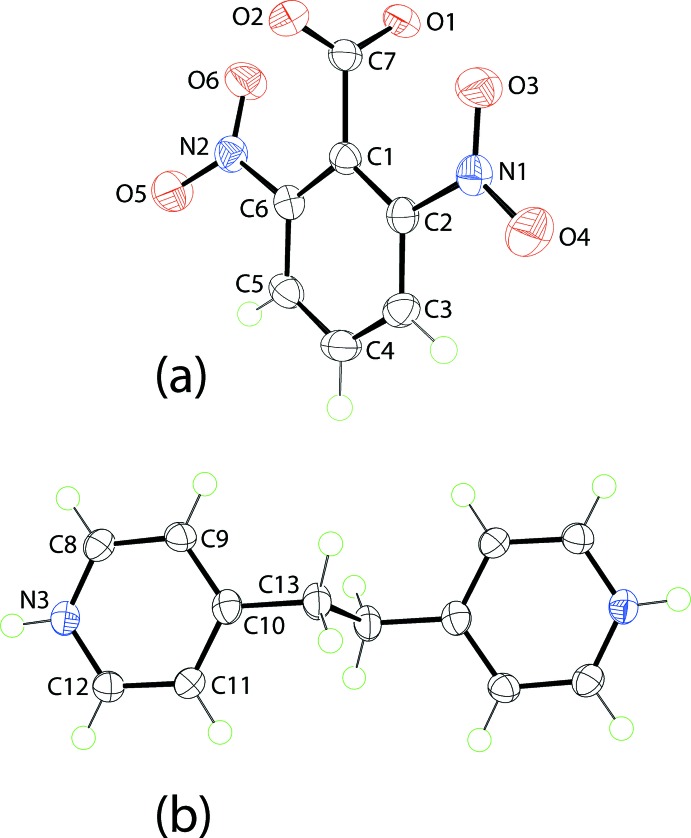
Molecular structures of the components of (I), showing atom-labelling scheme and displacement ellipsoids at the 70% probability level: (a) the 2,6-dinitrobenzoate anion, and (b) the 4-[2-(pyridin-1-ium-4-yl)ethyl]pyridin-1-ium dication (unlabelled atoms are related by the symmetry operation -x, 2 - y, 1-z).

**Fig. 2.**
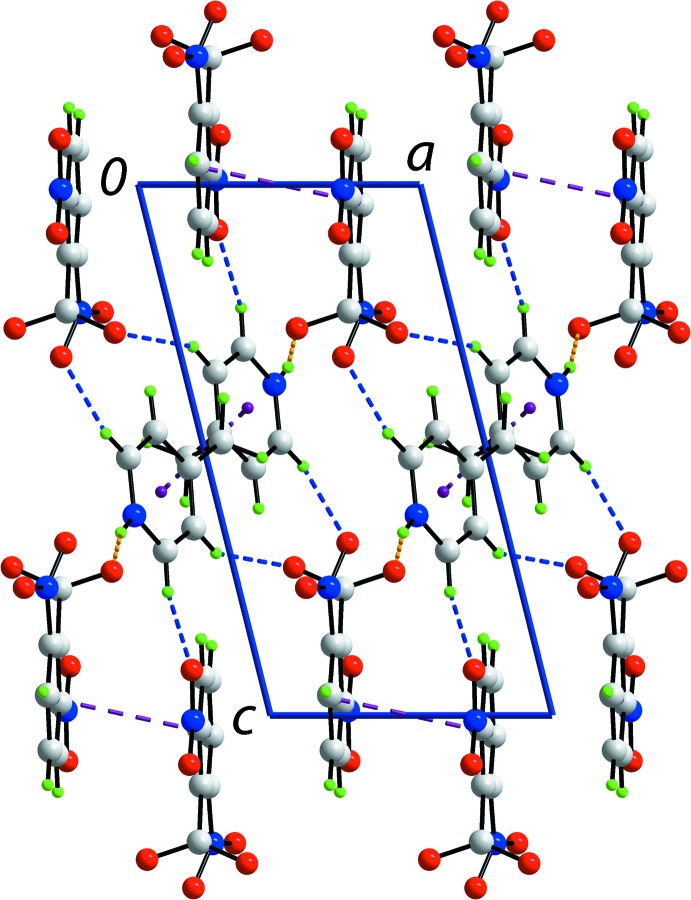
Unit-cell contents in (I) viewed down the b axis. The N—H···O, C—H···O and π—π interactions are shown as orange, blue and purple dashed lines, respectively.

## supplementary crystallographic information

### 1. Comment

The title salt, (I), was isolated as part of studies investigating the co-crystallization of 2,6-dinitrobenzoic acid and different pyridyl derivatives (Arman *et al.*, 2013).

The asymmetric unit of (I) comprises half of a 4-[2-(pyridin-1-ium-4-yl)ethyl]pyridin-1-ium dication, disposed about a centre of inversion, and a 2,6-dinitrobenzoate anion, Fig. 1. A kink about the ethylene bridge is evident in the dication with the C9—C10—C13—C13^i^ torsion angle being 104.1 (2)° [symmetry operation *i*: -*x*, 2 - *y*, 1 - *z*]. In the anion, the carboxylate is inclined to the benzene ring to which it is attached forming a dihedral angle of 85.45 (9)°, as is seen in the structures of related compounds, including that of 2,6-dinitrobenzoic acid (Arman *et al.*, 2013). One nitro group is co-planar and the other twisted out of the plane of the benzene ring to which they are attached, forming O3—N2—C2—C3 and O5—N1—C6—C5 torsion angles of 179.80 (14) and 20.2 (2)°. respectively.

The ions are connected into three-component aggregates by charge-assisted N—H···O hydrogen bonds, Table 1. These are connected by C—H···O and π—π [inter-centroid distance for centrosymmetrically related pyridinium rings = 3.6796 (14) Å for symmetry operation -*x*, 1 - *y*, 1 - *z*, and for centrosymmetrically related benzene rings = 3.7064 (14) Å for 1 - *x*, -*y*, -*z*] interactions into a three-dimensional architecture, Fig. 2.

### 2. Experimental

Crystals of (I) were obtained by the co-crystallization of 4,4'-bipyridylethane (Sigma Aldrich, 0.11 mmol) and 2,6-dinitrobenzoic acid (Sigma-Aldrich, 0.22 mmol) in methanol solution. Colourless crystals were obtained by slow evaporation. Melting point: 425–431 K. IR spectra (cm^-1^): 744(w), 849(*s*)(sh), 915(*m*), 1340(*m*), 1505(*m*), 1529(*s*), 1628(*m*)(sh), 1673(w), 3086(w)(br).

### 3. Refinement

The C-bound H-atoms were placed in calculated positions (C—H = 0.95–0.99 Å) and were included in the refinement in the riding model approximation with *U*_iso_(H) set to 1.2*U*_eq_(C). The N-bound H-atom was located in a difference Fourier map and refined with a distance restraint of N—H = 0.88±0.01 Å, and with *U*_iso_(H) = 1.2*U*_eq_(N).

### Crystal data

**Table:**

0.5C_12_H_14_N_2_^2+^·C_7_H_3_N_2_O_6_^−^	*Z* = 2
*M**_r_* = 304.24	*F*(000) = 314
Triclinic, *P*1	*D*_x_ = 1.581 Mg m^−^^3^
Hall symbol: -P 1	Mo *K*α radiation, λ = 0.71069 Å
*a* = 6.6916 (12) Å	Cell parameters from 3299 reflections
*b* = 8.3690 (17) Å	θ = 3.0–40.2°
*c* = 12.358 (3) Å	µ = 0.13 mm^−^^1^
α = 88.809 (12)°	*T* = 98 K
β = 76.322 (8)°	Chip, colourless
γ = 72.193 (9)°	0.36 × 0.12 × 0.07 mm
*V* = 639.2 (2) Å^3^	

### Data collection

**Table:**

Rigaku AFC12K/SATURN724 diffractometer	2538 reflections with *I* > 2σ(*I*)
Radiation source: fine-focus sealed tube	*R*_int_ = 0.035
Graphite monochromator	θ_max_ = 27.5°, θ_min_ = 3.0°
ω scans	*h* = −8→7
4421 measured reflections	*k* = −10→8
2915 independent reflections	*l* = −15→16

### Refinement

**Table:**

Refinement on *F*^2^	Primary atom site location: structure-invariant direct methods
Least-squares matrix: full	Secondary atom site location: difference Fourier map
*R*[*F*^2^ > 2σ(*F*^2^)] = 0.050	Hydrogen site location: inferred from neighbouring sites
*wR*(*F*^2^) = 0.124	H atoms treated by a mixture of independent and constrained refinement
*S* = 1.07	*w* = 1/[σ^2^(*F*_o_^2^) + (0.0536*P*)^2^ + 0.3505*P*] where *P* = (*F*_o_^2^ + 2*F*_c_^2^)/3
2915 reflections	(Δ/σ)_max_ < 0.001
202 parameters	Δρ_max_ = 0.33 e Å^−^^3^
1 restraint	Δρ_min_ = −0.28 e Å^−^^3^

### Special details

**Table:**

Geometry. All s.u.'s (except the s.u. in the dihedral angle between two l.s. planes) are estimated using the full covariance matrix. The cell s.u.'s are taken into account individually in the estimation of s.u.'s in distances, angles and torsion angles; correlations between s.u.'s in cell parameters are only used when they are defined by crystal symmetry. An approximate (isotropic) treatment of cell s.u.'s is used for estimating s.u.'s involving l.s. planes.
Refinement. Refinement of *F*^2^ against ALL reflections. The weighted *R*-factor *wR* and goodness of fit *S* are based on *F*^2^, conventional *R*-factors *R* are based on *F*, with *F* set to zero for negative *F*^2^. The threshold expression of *F*^2^ > 2σ(*F*^2^) is used only for calculating *R*-factors(gt) *etc*. and is not relevant to the choice of reflections for refinement. *R*-factors based on *F*^2^ are statistically about twice as large as those based on *F*, and *R*- factors based on ALL data will be even larger.

### Fractional atomic coordinates and isotropic or equivalent isotropic displacement parameters (Å^2^)

**Table:**

	*x*	*y*	*z*	*U*_iso_*/*U*_eq_	
O1	0.43537 (19)	0.16380 (14)	0.27124 (10)	0.0207 (3)	
O2	0.7856 (2)	0.08627 (15)	0.27881 (10)	0.0230 (3)	
O3	0.6692 (2)	0.30495 (15)	0.09243 (11)	0.0275 (3)	
O4	0.7613 (2)	0.25616 (17)	−0.08566 (11)	0.0323 (3)	
O5	0.7607 (3)	−0.43097 (15)	0.24069 (12)	0.0352 (4)	
O6	0.5746 (2)	−0.18948 (16)	0.32479 (11)	0.0304 (3)	
N1	0.6805 (2)	−0.27800 (17)	0.24086 (12)	0.0210 (3)	
N2	0.7206 (2)	0.21045 (17)	0.00970 (12)	0.0185 (3)	
C1	0.6929 (2)	−0.02772 (19)	0.13148 (13)	0.0158 (3)	
C2	0.7358 (2)	0.03113 (19)	0.02412 (13)	0.0163 (3)	
C3	0.7944 (3)	−0.0701 (2)	−0.07317 (14)	0.0193 (3)	
H3	0.8218	−0.0241	−0.1441	0.023*	
C4	0.8122 (3)	−0.2389 (2)	−0.06528 (14)	0.0222 (4)	
H4	0.8515	−0.3095	−0.1310	0.027*	
C5	0.7727 (3)	−0.3049 (2)	0.03835 (14)	0.0204 (3)	
H5	0.7844	−0.4206	0.0445	0.024*	
C6	0.7156 (3)	−0.19921 (19)	0.13322 (14)	0.0172 (3)	
C7	0.6353 (3)	0.08483 (19)	0.23831 (13)	0.0168 (3)	
N3	0.3059 (2)	0.44597 (17)	0.37677 (12)	0.0181 (3)	
H3N	0.369 (3)	0.3421 (15)	0.3443 (16)	0.022*	
C8	0.2755 (3)	0.4803 (2)	0.48557 (14)	0.0186 (3)	
H8	0.3349	0.3942	0.5308	0.022*	
C9	0.1587 (3)	0.63957 (19)	0.53319 (13)	0.0182 (3)	
H9	0.1402	0.6635	0.6104	0.022*	
C10	0.0682 (3)	0.76502 (19)	0.46686 (14)	0.0177 (3)	
C11	0.1067 (3)	0.7261 (2)	0.35320 (14)	0.0185 (3)	
H11	0.0501	0.8096	0.3057	0.022*	
C12	0.2273 (3)	0.5659 (2)	0.31021 (14)	0.0193 (3)	
H12	0.2553	0.5398	0.2326	0.023*	
C13	−0.0661 (3)	0.93814 (19)	0.51519 (14)	0.0197 (3)	
H13A	−0.1124	0.9362	0.5973	0.024*	
H13B	−0.1972	0.9759	0.4857	0.024*	

### Atomic displacement parameters (Å^2^)

**Table:**

	*U*^11^	*U*^22^	*U*^33^	*U*^12^	*U*^13^	*U*^23^
O1	0.0211 (6)	0.0170 (6)	0.0209 (6)	−0.0035 (4)	−0.0020 (5)	−0.0026 (4)
O2	0.0256 (6)	0.0242 (6)	0.0202 (6)	−0.0065 (5)	−0.0090 (5)	−0.0016 (5)
O3	0.0425 (8)	0.0181 (6)	0.0238 (7)	−0.0123 (5)	−0.0075 (6)	0.0014 (5)
O4	0.0449 (8)	0.0296 (7)	0.0206 (7)	−0.0132 (6)	−0.0033 (6)	0.0102 (5)
O5	0.0588 (10)	0.0139 (6)	0.0325 (8)	−0.0068 (6)	−0.0161 (7)	0.0047 (5)
O6	0.0387 (8)	0.0234 (7)	0.0198 (7)	−0.0035 (6)	0.0027 (5)	0.0028 (5)
N1	0.0243 (7)	0.0168 (7)	0.0233 (8)	−0.0067 (5)	−0.0079 (6)	0.0035 (5)
N2	0.0172 (7)	0.0192 (7)	0.0194 (7)	−0.0068 (5)	−0.0038 (5)	0.0045 (5)
C1	0.0135 (7)	0.0161 (7)	0.0168 (8)	−0.0035 (6)	−0.0030 (6)	0.0002 (6)
C2	0.0146 (7)	0.0164 (7)	0.0181 (8)	−0.0049 (6)	−0.0043 (6)	0.0026 (6)
C3	0.0160 (7)	0.0251 (8)	0.0153 (8)	−0.0051 (6)	−0.0026 (6)	0.0004 (6)
C4	0.0191 (8)	0.0262 (9)	0.0194 (8)	−0.0043 (7)	−0.0039 (6)	−0.0066 (6)
C5	0.0192 (8)	0.0162 (7)	0.0248 (9)	−0.0036 (6)	−0.0058 (6)	−0.0031 (6)
C6	0.0160 (7)	0.0160 (7)	0.0198 (8)	−0.0047 (6)	−0.0051 (6)	0.0019 (6)
C7	0.0225 (8)	0.0127 (7)	0.0144 (7)	−0.0055 (6)	−0.0027 (6)	0.0018 (5)
N3	0.0176 (7)	0.0151 (6)	0.0201 (7)	−0.0050 (5)	−0.0017 (5)	0.0002 (5)
C8	0.0190 (8)	0.0171 (8)	0.0200 (8)	−0.0067 (6)	−0.0039 (6)	0.0035 (6)
C9	0.0217 (8)	0.0186 (8)	0.0145 (7)	−0.0081 (6)	−0.0027 (6)	0.0015 (6)
C10	0.0175 (7)	0.0159 (7)	0.0201 (8)	−0.0080 (6)	−0.0017 (6)	0.0006 (6)
C11	0.0218 (8)	0.0165 (7)	0.0178 (8)	−0.0066 (6)	−0.0052 (6)	0.0029 (6)
C12	0.0229 (8)	0.0183 (8)	0.0165 (8)	−0.0070 (6)	−0.0035 (6)	0.0011 (6)
C13	0.0217 (8)	0.0147 (7)	0.0205 (8)	−0.0061 (6)	−0.0001 (6)	−0.0012 (6)

### Geometric parameters (Å, º)

**Table:**

O1—C7	1.267 (2)	C5—H5	0.9500
O2—C7	1.228 (2)	N3—C8	1.335 (2)
O3—N2	1.2206 (18)	N3—C12	1.345 (2)
O4—N2	1.2258 (19)	N3—H3N	0.897 (9)
O5—N1	1.2273 (19)	C8—C9	1.383 (2)
O6—N1	1.2207 (19)	C8—H8	0.9500
N1—C6	1.473 (2)	C9—C10	1.397 (2)
N2—C2	1.483 (2)	C9—H9	0.9500
C1—C6	1.397 (2)	C10—C11	1.394 (2)
C1—C2	1.402 (2)	C10—C13	1.500 (2)
C1—C7	1.539 (2)	C11—C12	1.377 (2)
C2—C3	1.391 (2)	C11—H11	0.9500
C3—C4	1.384 (2)	C12—H12	0.9500
C3—H3	0.9500	C13—C13^i^	1.543 (3)
C4—C5	1.384 (2)	C13—H13A	0.9900
C4—H4	0.9500	C13—H13B	0.9900
C5—C6	1.388 (2)		
			
O6—N1—O5	123.53 (15)	O1—C7—C1	113.71 (14)
O6—N1—C6	119.05 (14)	C8—N3—C12	120.98 (14)
O5—N1—C6	117.43 (14)	C8—N3—H3N	123.2 (13)
O3—N2—O4	123.22 (14)	C12—N3—H3N	115.6 (13)
O3—N2—C2	118.95 (13)	N3—C8—C9	120.80 (15)
O4—N2—C2	117.82 (14)	N3—C8—H8	119.6
C6—C1—C2	114.17 (14)	C9—C8—H8	119.6
C6—C1—C7	122.75 (14)	C8—C9—C10	119.46 (15)
C2—C1—C7	123.03 (14)	C8—C9—H9	120.3
C3—C2—C1	123.70 (15)	C10—C9—H9	120.3
C3—C2—N2	116.29 (14)	C11—C10—C9	118.29 (15)
C1—C2—N2	120.01 (14)	C11—C10—C13	120.17 (15)
C4—C3—C2	119.06 (15)	C9—C10—C13	121.55 (15)
C4—C3—H3	120.5	C12—C11—C10	119.58 (15)
C2—C3—H3	120.5	C12—C11—H11	120.2
C5—C4—C3	120.03 (15)	C10—C11—H11	120.2
C5—C4—H4	120.0	N3—C12—C11	120.82 (15)
C3—C4—H4	120.0	N3—C12—H12	119.6
C4—C5—C6	118.91 (15)	C11—C12—H12	119.6
C4—C5—H5	120.5	C10—C13—C13^i^	110.09 (16)
C6—C5—H5	120.5	C10—C13—H13A	109.6
C5—C6—C1	124.13 (15)	C13^i^—C13—H13A	109.6
C5—C6—N1	116.20 (14)	C10—C13—H13B	109.6
C1—C6—N1	119.67 (14)	C13^i^—C13—H13B	109.6
O2—C7—O1	129.53 (15)	H13A—C13—H13B	108.2
O2—C7—C1	116.76 (14)		
			
C6—C1—C2—C3	0.5 (2)	O6—N1—C6—C5	−159.79 (16)
C7—C1—C2—C3	178.01 (14)	O5—N1—C6—C5	20.2 (2)
C6—C1—C2—N2	−179.28 (13)	O6—N1—C6—C1	20.9 (2)
C7—C1—C2—N2	−1.7 (2)	O5—N1—C6—C1	−159.13 (16)
O3—N2—C2—C3	179.80 (14)	C6—C1—C7—O2	84.16 (19)
O4—N2—C2—C3	−0.4 (2)	C2—C1—C7—O2	−93.16 (19)
O3—N2—C2—C1	−0.4 (2)	C6—C1—C7—O1	−96.06 (18)
O4—N2—C2—C1	179.36 (15)	C2—C1—C7—O1	86.61 (19)
C1—C2—C3—C4	0.0 (2)	C12—N3—C8—C9	1.2 (2)
N2—C2—C3—C4	179.77 (14)	N3—C8—C9—C10	1.2 (2)
C2—C3—C4—C5	−0.2 (2)	C8—C9—C10—C11	−2.5 (2)
C3—C4—C5—C6	−0.1 (3)	C8—C9—C10—C13	177.92 (14)
C4—C5—C6—C1	0.6 (3)	C9—C10—C11—C12	1.5 (2)
C4—C5—C6—N1	−178.67 (15)	C13—C10—C11—C12	−178.94 (15)
C2—C1—C6—C5	−0.8 (2)	C8—N3—C12—C11	−2.3 (2)
C7—C1—C6—C5	−178.33 (15)	C10—C11—C12—N3	0.9 (2)
C2—C1—C6—N1	178.46 (14)	C11—C10—C13—C13^i^	−75.5 (2)
C7—C1—C6—N1	0.9 (2)	C9—C10—C13—C13^i^	104.1 (2)

Symmetry code: (i) −*x*, −*y*+2, −*z*+1.

### Hydrogen-bond geometry (Å, º)

**Table:**

*D*—H···*A*	*D*—H	H···*A*	*D*···*A*	*D*—H···*A*
N3—H3n···O1	0.90 (1)	1.64 (2)	2.5240 (19)	166 (2)
C8—H8···O6^ii^	0.95	2.50	3.436 (2)	169
C11—H11···O2^iii^	0.95	2.52	3.377 (2)	150
C12—H12···O4^iv^	0.95	2.46	3.118 (2)	126

Symmetry codes: (ii) −*x*+1, −*y*, −*z*+1; (iii) *x*−1, *y*+1, *z*; (iv) −*x*+1, −*y*+1, −*z*.
